# Ectopic Oral Tonsillar Tissue: A Case Series with Bilateral and Solitary Presentations and a Review of the Literature

**DOI:** 10.1155/2015/518917

**Published:** 2015-01-14

**Authors:** Masashi Kimura, Toru Nagao, Terumi Saito, Saman Warnakulasuriya, Hiroyuki Ohto, Akihito Takahashi, Kanji Komaki, Yoshiyuki Naganawa

**Affiliations:** ^1^Department of Dentistry Oral and Maxillofacial Surgery, Ogaki Municipal Hospital, 4-86 Minaminokawa, Ogaki, Gifu 503-8502, Japan; ^2^Department of Oral and Maxillofacial Surgery and Stomatology, Okazaki City Hospital, 3-1 Goshoai, Koryuji-cho, Okazaki, Aichi 444-8553, Japan; ^3^Department of Oral Medicine, King's College London Dental Institute, WHO Collaborating Centre for Oral cancer/Precancer, Bessemer Road, London SE5 9RS, UK; ^4^Department of Oral and Maxillofacial Surgery, Yokkaichi Municipal Hospital, Yokkaichi, Mie 510-8567, Japan

## Abstract

An ectopic tonsil is defined as tonsillar tissue that develops in areas outside of the four major tonsil groups: the palatine, lingual, pharyngeal, and tubal tonsils. The occurrence of tonsillar tissue in the oral cavity in ectopic locations, its prevalence, and its developmental mechanisms that belong to its formation remain unclear. In this report, we describe a rare case of bilateral symmetric ectopic oral tonsillar tissue located at the ventral surface of the tongue along with two solitary cases arising from the floor of the mouth. The role of immune system and its aberrant response leading to ectopic deposits desires further studies. As an ectopic tonsil may simulate a benign soft tissue tumor, this case series highlights the importance of this entity in our clinical differential diagnosis of oral soft tissue masses.

## 1. Introduction

The tonsils form part of a circular band of adenoid tissue known as Waldeyer's ring, which guard the opening of the digestive and respiratory tracts. This circular band is comprised of four major tonsil groups: the palatine, lingual, pharyngeal, and tubal tonsils. An ectopic tonsil is tonsillar tissue that develops in areas outside of these regions. The existence of ectopic oral tonsils was described by Knapp in 1970 [1]. It was shown that such structures, resembling pharyngeal and other tonsils, can be found within the oral cavity.

Ectopic tonsils have been reported in different anatomic locations of the oral cavity, for example, on the floor of the mouth [1–6], ventral surface of the tongue [1, 2, 4], and soft palate [1, 2], and in other parts of the aerodigestive tracts, for example, larynx [7], hypopharynx [8], nasal septum [9], or in the orbit [10] (Table 1). Collection of tonsillar tissue in ectopic sites can cause diagnostic confusion; however, none of the reported cases have been described with a bilateral presentation and/or symmetrically such as that found in the oropharynx.

Here we report a rare case of bilateral symmetric ectopic oral tonsillar tissue observed on the ventral surface of the tongue and two other solitary cases arising from floor of the mouth along with a review of the literature.

## 2. Case Presentations

### 2.1. Case 1

**Figure 1:**
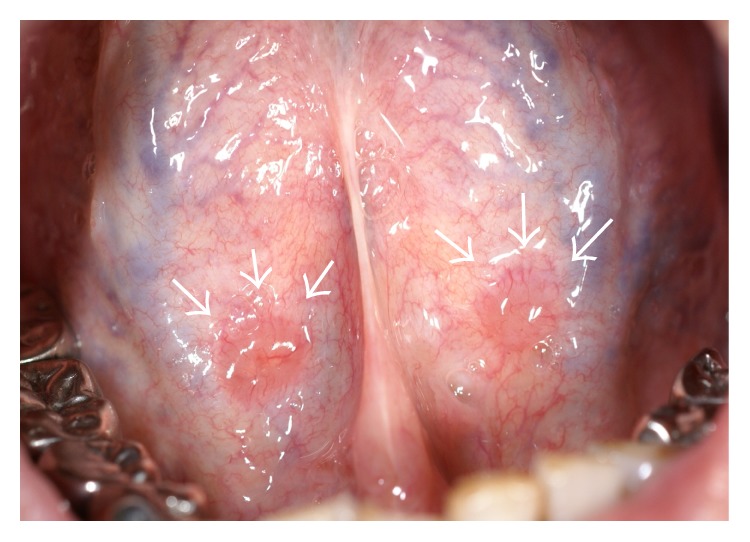
Clinical findings of Case 1. Small, bilaterally symmetric masses on the ventral surface of the tongue (arrows).

**Figure 2:**
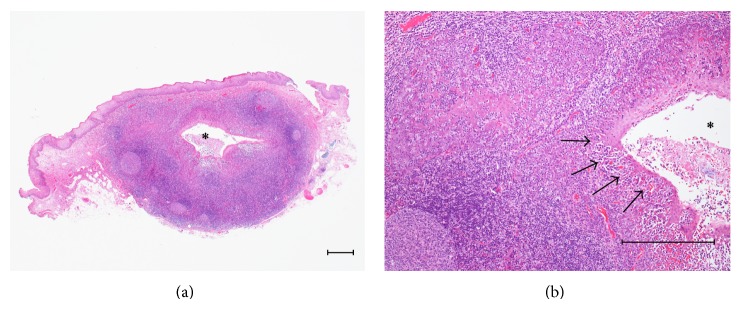
(a) Histopathological findings of Case 1. Germinal center, lymphoid tissue, and a crypt (^*^) are seen (Hematoxylin-Eosin (HE), scale bar = 250 *μ*m). (b) Lymphoepithelial symbiosis in the crypt is seen (arrows) (HE, scale bar = 250 *μ*m).

A 53-year-old Japanese male, referred by his general dental practitioner, presented with small, bilaterally symmetric masses on the ventral surface of the tongue, noticed during a routine dental examination 2 months ago. The areas affected were painless and remained unchanged in size over the previous 2 months. Intraoral examination revealed hard masses of 8 mm diameter (right) and 6 mm diameter (left) on the ventral surface of the tongue (Figure 1). The surface covering of these masses was slightly red and was hard on palpation. Clinically, a small pit was evident at the tip of both masses; a provisional diagnosis of bilateral benign tumors of salivary origin was made. An excision biopsy of the mass on the right side was subsequently performed under local anesthesia. The mass was easily resected and the postoperative course was uneventful. Histopathological findings showed a germinal center, lymphoid tissue, and lymphoepithelial symbiosis in the crypt (Figure 2). Although the bilateral symmetric ectopic oral tonsillar tissue arising from this region has not been reported elsewhere to our knowledge, clinicopathological characteristics were similar to two other cases (Cases 2 and 3) of solitary origin presented later in our clinic (Table 2).

### 2.2. Case 2

**Figure 3:**
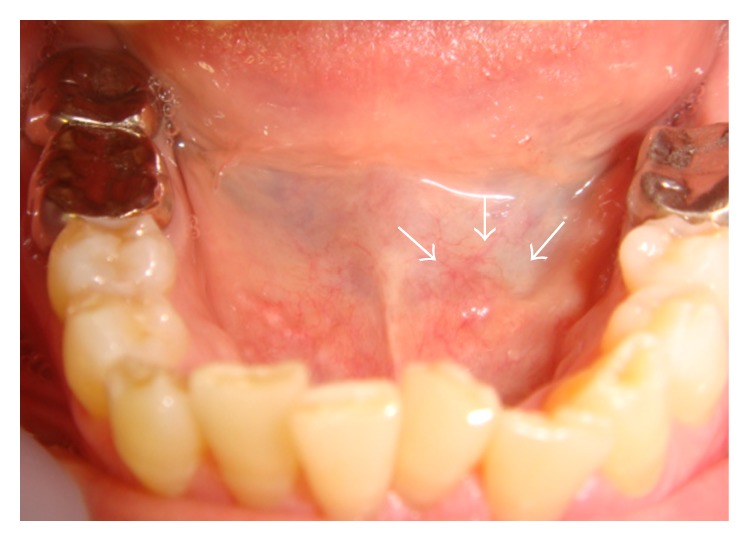
Clinical findings of Case 2. A small mass on the left side of the floor of the mouth (arrows). The mass was slightly red.

A 63-year-old Japanese female presented at our hospital with a small swelling on the left side of the floor of the mouth. She first noticed this lump 10 days previously. The affected area was painless and its size remained unchanged. Intraoral examination revealed a well-circumscribed mass (5 mm diameter) on the left side of the floor of the mouth (Figure 3). The mass was slightly red and hard on palpation and was clinically diagnosed as a benign salivary tumor of the floor of the mouth. It was resected under local anesthesia and at excision was found to be encapsulated and appeared fairly close to the sublingual salivary gland. However, it was completely detached from the gland by its own capsule. The postoperative course was uneventful. Histopathology revealed characteristic features of a tonsil with a germinal center, a mass of lymphoid tissue, and a crypt with lymphoepithelial symbiosis. These findings were suggestive of ectopic tonsillar tissue (Table 2).

### 2.3. Case 3

**Figure 4:**
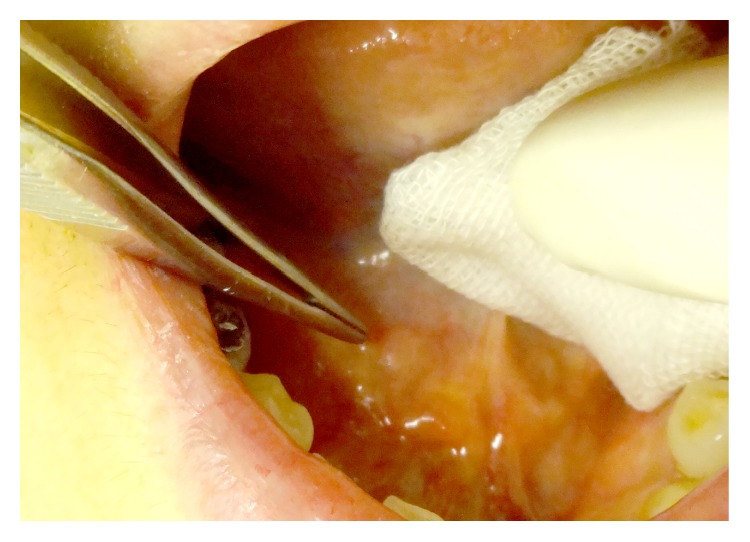
Clinical findings of Case 3. A small mass covered by intact normal-appearing mucosa on the right side of the floor of the mouth.

A 38-year-old Japanese female visited our clinic complaining of a small painless lump on the right side of the floor of the mouth. She first noticed this lesion 2 days ago. Intraoral examination revealed a well-circumscribed mass (6 mm diameter) covered by intact normal-appearing mucosa (Figure 4). The mass was soft on palpation and was clinically diagnosed as a mucocele of the floor of the mouth. It was resected under local anesthesia and at excision it was completely detached from the sublingual salivary gland and Wharton's duct by its own capsule. The postoperative course was uneventful. Pathological characteristics were similar to the earlier described cases (Table 2).

## 3. Discussion

Ectopic tonsils are comprised of a single or branched crypts containing lymphoid follicles lined with stratified squamous epithelium. In Table 1, we present single cases and case series of ectopic tonsils. A literature search was conducted in August 2014 using the electronic databases PubMed and Scopus and hand-searching using the search term of ectopic tonsil. The search was restricted to published articles containing clinicopathological features. Furthermore, search parameter was also set to select literature restricted to English language only.

As a result only 62 cases have been reported in the English language. The most frequently affected area is the floor of the mouth (59% of cases), followed by the soft palate (24.6%) and ventral surface of the tongue (9.8%). In the clinical findings, the size of the lesions ranged from 3 to 28 mm with rounded shape, and the surface covering of the lesions was occasionally and slightly red. Therefore, they may cause diagnostic confusion, especially when found around the floor of the mouth. It may be misdiagnosed as tumors that arise from the sublingual gland.

According to Knapp, lymphoepithelial cyst that originates from lymphoid tissue following obstruction of these crypts also may have similar presentation. Clinically, these lesions appear yellowish and comprise a cystic cavity that appears as a dilated crypt lined with a stratified squamous epithelium [2]. In the three cases presented here, although several serial sections of the specimens were examined, there was no evidence of cyst formation or crypt obstruction. On the basis of these histopathological findings, the authors diagnosed these masses as ectopic tonsils. According to Patel et al. [4] inflamed ectopic tonsils may swell and become tender, thus requiring resection. Usually, however, ectopic oral tonsils remain asymptomatic and can be left untreated, but surgical exploration is indicated to establish a tissue diagnosis [4]. In Case 1, excisional biopsy of one mass led to a histopathological diagnosis of ectopic tonsillar tissue. Thus, the need for surgical resection of the contralateral lesion was avoided.

The pathogenesis of ectopic tonsils in this region remains unclear. Lymphoid tissue is also found in fetal salivary glands, and occasionally remnants of lymphoid tissue are found in adult salivary glands [11]. The masses in Cases 2 and 3 appeared close to the sublingual gland but were completely separated from the salivary tissues, whereas the masses in Case 1 were placed distant from the salivary tissues, and thus the origins of these masses remained obscure. It is reported that ectopic tonsillar tissue in the nasal septum may result from persistent infection [9]. However, in Case 1, because the masses were bilateral and symmetrical, the etiology was not considered to be reactive lymphoid hyperplasia.

These cases reported by us highlight the possibility of ectopic oral tonsillar tissue and raise the need to consider them when making a differential diagnosis of soft tissue lumps found on the floor of the mouth and/or the ventral surface of the tongue. Further cadaveric study is required to clarify the presence of ectopic tonsillar tissue on these anatomical sites, particularly with regard to its developmental mechanisms, and to assess its prevalence and to study the clinical significance of the immune system and its response.

Ectopic tonsils appear to occur more frequently than are generally recognized, probably because they are usually asymptomatic and are thus easily overlooked. We have described these three cases of ectopic tonsils to propose that clinicians may consider inclusion of this entity in the clinical differential diagnosis, often not encountered in reference text books in oral medicine and pathology.

## Figures and Tables

**Table 1:** Summary of ectopic tonsils reported in the literatures.

Author	Year	Anatomic location	Number of cases		Clinical presentation		Microscopic findings
					Clinical features	Lesion size (mm)	
		Floor of the mouth	32		Firm, nodular, pale pink, and up to 10 mm	*←*	**Hypertrophic oral tonsil** (i) Numerous enlarged lymphoid follicles with germinal centers (ii) Single or a branched crypt which was lined with stratified squamous epithelium
Knapp [2]	1970	Ventral surface of the tongue	5	52 total	Slightly compressible, yellowish “cystic” mass, creamy or cheese-like discharge, and up to 10 mm	*←*	**Tonsillar pseudocyst** (i) The lesion consisted of a cystic cavity which represented a dilated crypt lined with stratified squamous epithelium
		Soft palate	15		Red, firm rounded nodule, and from 1 to 3 mm	*←*	**Hyperemic oral tonsil** (i) It showed a prominent hyperemia of the tonsillar and peritonsillar blood vessels

Wolter and Roosenberg [10]	1977	Orbit	1		A smooth surface, an oval shape, and a rubber-like consistency	24 × 15 × 10	(i) Many primary lymphoid nodules with germinal centers

Paslin [5]	1980	Floor of the mouth	1		Oval, pink, lucent, rounded, and firm papule on the sublingual fold just to the right of the frenulum.	3 × 3	(i) Circumscribed masses of lymphoid cells forming germinal centers surrounding the central crypt of stratified squamous epithelium

Pellettiere et al. [7]	1980	Larynx	1		Firm and freely movable and covered by normal appearing, smooth, and intact mucosa	15	(i) Moderately well delineated germinal center

Furukawa et al. [9]	1983	Nasal septum	1		Firm and greyish-white mass	28 × 22 × 14	(i) The surface epithelium of the tumour was fibrous tissue covered with squamous cells which invaginated into the lymphoid tissue producing crypts surrounded by lymphoid follicles

Mogi [3]	1991	Floor of the mouth	1		Small, dark red, and soft tumor with no tender	6 × 3 × 3	(i) A germinal center surrounded by fibrous tissue invaded by squamous epithelium

Patel et al. [4]	2004	Floor of the mouth	1		Three small, red, and circular lesions in the mucosa of the floor of the mouth	3	(i) Aggregation of lymphoid tissue within the lamina propria (ii) Well-defined lymphoid follicles
		Ventral surface of the tongue	1		White, soft, and nontender mucosal nodule of the frenum of the ventral surface of the tongue	4	(i) A focus of lymphoid tissue including follicles with well-formed germinal centers (ii) A cystic lesion lined with stratified squamous epithelium filled with keratinous debris

Baba et al. [8]	2010	Hypopharynx	1		Smooth mucosal swelling in the right pyriform recess	No mention	(i) Germinal center, lymphoid tissue, and crypt involving lymphoepithelial symbiosis

Kashima et al. [6]	2012	Floor of the mouth	1		Well-circumscribed, smooth, round, painless, swelling covered by intact normal-appearing mucosa	4	(i) Abundant reactive lymphoid aggregates with well-formed germinal centers (ii) A nondilated central crypt lined with stratified squamous epithelium and containing desquamated epithelial cells (iii) Keratin debris in a central lacuna-like space

Present cases (Kimura et al.)	2014	Ventral surface of the tongue	1		Well-circumscribed, slightly red, hard on palpation, and bilateral presentation A small pit was evident at the tip (Case 1)	8/6	Shown in Table 2
		Floor of the mouth	2		Well-circumscribed, slightly red, and hard on palpation (Case 2)	5	
					Well-circumscribed and soft on palpation mass covered by normal mucosa (Case 3)	6	

**Table 2:** Clinicopathological characteristics of three cases of ectopic tonsils.

	Case number		
	1	2	3
Gender	Male	Female	Female
Age	53	63	38
Localization	Ventral surface of the tongue	Floor of the mouth	Floor of the mouth
Number of lesions	Bilateral	Solitary	Solitary
Lesion size (mm)	8/6	5	6
Color of oral mucosa	Slightly red	Slightly red	Normal
Palpation	Hard	Hard	Soft
Clinical diagnosis	Benign salivary tumor	Benign salivary tumor	Mucocele
Histopathological findings			
Crypt architecture	+	+	+
Encapsulation	+	+	+
Lymphoepithelial symbioses	+	+	+
Lymphoid follicle	+	+	+
Crypt obstruction	−	−	−
Cyst formation	−	−	−
